# Editorial for the Special Issue “Genetics and Molecular Breeding in Fisheries and Aquaculture”

**DOI:** 10.3390/genes17010033

**Published:** 2025-12-30

**Authors:** Lei Liu, Kai Zhang, Xuedi Du

**Affiliations:** 1 School of Marine Sciences, Ningbo University, Ningbo 315832, China; 2 College of Marine Science and Engineering, Nanjing Normal University, Nanjing 210023, China; zkkaka@126.com; 3 College of Animal Science and Technology, Yangzhou University, Yangzhou 225009, China; xddu@yzu.edu.cn

The burgeoning global population and the increasing recognition of seafood’s nutritional value place unprecedented pressure on aquatic food systems. While aquaculture stands as the fastest-growing food production sector, its sustainable intensification is challenged by disease, environmental variability, feed sustainability, and the need to genetically improve a diversity of species. The convergence of genetics and molecular biology with traditional breeding practices represents a powerful paradigm that enables us to address these challenges. This Special Issue, “Genetics and Molecular Breeding in Fisheries and Aquaculture”, presents a collection of pioneering studies that exemplify this synergy. The contributions span from the development of foundational genetic tools to the elucidation of molecular mechanisms underlying key economic traits, providing a roadmap for the future of precision aquaculture. 

A critical first step in any modern breeding program is the establishment of robust genetic management systems. The paper by Jiang et al. [1] on the sea urchin *Strongylocentrotus intermedius* addresses this directly. By developing a microsatellite (SSR-seq) based parentage assignment method with over 90% identification success, the authors provide a practical tool for managing breeding cohorts. This is vital for preventing inbreeding, maintaining genetic diversity, and enabling accurate pedigree tracking in selective breeding programs—a fundamental requirement for long-term genetic gain.

Expanding this theme to a population level, He et al. [2] employ a genotyping-by-sequencing (GBS) approach to analyze the genome-wide population structure of the swimming crab *Charybdis feriata*. Their discovery of high genetic connectivity along China’s coast, with a subtle distinctiveness in the Zhoushan population, provides crucial data for sustainable fishery management and the selection of genetically diverse broodstock for nascent aquaculture efforts.

## 1. Decoding Complex Traits: From QTL Mapping to Molecular Pathways

Moving beyond description to causation, several papers delve into the genetic and molecular basis of commercially important traits. Zhang et al. [3] conduct a fine-mapping exercise for alkaline tolerance in Crucian Carp, identifying specific quantitative trait loci (QTLs) using genome-wide SNP markers. This work paves the way for marker-assisted selection to develop strains capable of thriving in challenging water conditions, a growing concern in inland aquaculture.

At the transcriptomic and proteomic levels, Du et al. [4] investigate the molecular underpinnings of heterosis in hybrid porgy. By analyzing the *TGF-β1*/*Smads* signaling pathway, they demonstrate significant expression changes in hybrids compared to their parental species (*Acanthopagrus schlegelii* and *Pagrus major*). This provides a mechanistic glimpse into hybrid vigor, suggesting that the superior growth of hybrids is orchestrated by complex regulatory networks, opening new avenues for research into predicting and exploiting heterosis.

## 2. Enhancing Resilience to Biotic and Abiotic Stresses

A prominent theme in this issue is the focus on health and environmental resilience. Multiple studies employ advanced molecular techniques to understand and improve stress tolerance. Chen et al. [5] identify and characterize key elements of the necroptosis pathway (RIPK and MLKL) in the sea cucumber *Holothuria leucospilota*. Their findings on tissue-specific and pathogen-responsive expression patterns offer new insights into the innate immune system of this valuable echinoderm, identifying potential candidates for breeding disease-resistant lines.

Similarly, Zhang et al. [6] conduct a comprehensive analysis of the HSP70 and HSP90 gene families in the pufferfish *Takifugu fasciatus*. They document distinct expression profiles under cold stress and different pathogen challenges, highlighting the versatile role of heat shock proteins in environmental adaptation and immunity. This gene family characterization is a vital resource for future studies on stress resilience in this species.

A standout contribution in this area is represented by a series of papers on the Australasian snapper (*Chrysophrys auratus*) by Bentley-Hewitt et al. In [7], they developed a novel NanoString gene panel to rapidly assess stress and immune responses to acute temperature shifts. This work is elegantly extended by applying a similar panel to chronic temperature exposure [8] and tracking gene expression during larval development [9]. Collectively, these studies not only identify key biomarker genes for thermal tolerance but also validate a non-lethal (fin clip) method for predicting internal health status. This has direct applications in selecting resilient broodstock and optimizing aquaculture environments.

## 3. Supporting Domestication and Nutritional Adaptation

The successful domestication of new species often hinges on understanding and manipulating feeding behavior. Wang et al. [10] explored the role of the *scd1* gene in the feeding habit transformation of mandarin fish (*Siniperca chuatsi*). Their finding that *scd1* expression is significantly upregulated in the livers of fish adapted to artificial diets points to a genetic component in feed adaptation. This molecular insight is invaluable for breeding programs aimed at domesticating carnivorous species that are traditionally reliant on live prey, thereby improving sustainability and reducing production costs.

## 4. Conclusions and Future Perspectives

The research compiled in this Special Issue clearly illustrates the transformative impact of genetics and molecular biology on aquaculture. We are witnessing a shift from a phenotypic to a genotypic era, where breeding decisions are informed by genomic parentage, precise QTLs, and an understanding of complex molecular pathways. The future of this field lies in the integration of these tools. The gene panels and biomarker identification studies in snappers demonstrate a path toward genomic selection for complex traits like multi-stress resilience, while the foundational resources developed for sea urchins, crabs, and fishes will enable genome-wide association studies and accelerate genetic progress. Furthermore, the mechanistic insights into heterosis and feeding adaptation provide a deeper biological understanding that can make breeding programs more predictive and efficient.

However, translating this research into industry-wide practice requires addressing challenges related to cost, bioinformatics capacity, and the conservation of genetic diversity. As we move forward, the integration of emerging technologies like genome editing and epigenetics with the approaches showcased in this Special Issue will further empower researchers to achieve these goals. The studies in this Special Issue collectively underscore that a sustainable and productive blue revolution will be, at its core, a genetic revolution. By continuing to build on these foundations, we can ensure that aquaculture fulfills its potential as a key solution to global food security.

## References

[1] Jiang X., Liu L., Guo H., Liu P., Tian W., Ou F., Ding J., Zhang W., Chang Y. (2024). Establishment of Parentage Identification Method for Sea Urchin *Strongylocentrotus intermedius* Based on SSR-seq Technology. Genes.

[2] He J., Wu J., Wan L., Xu W., Yang T. (2024). Genome-Wide Genetic Diversity and Population Structure of *Charybdis feriata* (Crustacea, Decapoda, and Portunidae) Along the Southeast Coast of China Inferred from Genotyping-by-Sequencing (GBS) Approach. Genes.

[3] Zhang L., Su B., Huang J., Zhang L., Chang Y., Hu G. (2024). Fine Mapping of QTLs for Alkaline Tolerance in Crucian Carp (*Carassius auratus*) Using Genome-Wide SNP Markers. Genes.

[4] Du X., Zhao Y., Li J., Xie W., Lyu L., Chen S., Jia C., Yan J., Li P. (2024). Expression Patterns of TGF-β1, TβR-I, TβR-II, and Smad2 Reveal Insights into Heterosis for Growth of Hybrid Offspring between *Acanthopagrus schlegelii* and *Pagrus major*. Genes.

[5] Chen R., Huang Q., Rao Y., Wang J., Yu R., Peng S., Huang K., Huang Y., Zhu X., Tang D. (2024). Genomic and Transcriptional Analysis of the Necroptosis Pathway Elements RIPK and MLKL in Sea Cucumber, Holothuria leucospilota. Genes.

[6] Zhang W., Qian Z., Ji J., Wang T., Yin S., Zhang K. (2024). Characterization of HSP70 and HSP90 Gene Family in *Takifugu fasciatus* and Their Expression Profiles on Biotic and Abiotic Stresses Response. Genes.

[7] Bentley-Hewitt K., Flammensbeck C.K., Crowhurst R.N., Hedderley D.I., Wellenreuther M. (2024). Development of a Novel Stress and Immune Gene Panel for the Australasian Snapper (*Chrysophrys auratus*). Genes.

[8] Bentley-Hewitt K., Hedderley D.I. (2024). Assessment of a Novel Stress and Immune Gene Panel on the Development of Australasian Snapper (*Chrysophrys auratus*) Larvae. Genes.

[9] Bentley-Hewitt K., Flammensbeck C.K., Hedderley D.I., Wellenreuther M. (2025). Assessment of Stress and Immune Gene Expression in Australasian Snapper (*Chrysophrys auratus*) Exposed to Chronic Temperature Change. Genes.

[10] Wang J., Zhang L., Gao X., Sun Y., Zhao C., Gao X., Wu C. (2024). Molecular Cloning of the scd1 Gene and Its Expression in Response to Feeding Artificial Diets to Mandarin Fish (*Siniperca chuatsi*). Genes.

